# Freshwater Algae Biostimulant in Mitigating Impacts of Saline Irrigation on Onions

**DOI:** 10.3390/plants14101559

**Published:** 2025-05-21

**Authors:** Jean Carlos Nogueira, Jefferson dos Santos Gomes Calaça, Carla Veronica Barbosa de Souza Gomes, Luiz Emanuel Callou Menezes, José Raliuson Inácio Silva, Alexandre Maniçoba da Rosa Ferraz Jardim, Luiz Guilherme Medeiros Pessoa, João Henrique Barbosa da Silva, Ramon Freire da Silva, Thiago Jardelino Dias, Genival Barros Júnior

**Affiliations:** 1 Postgraduate Program in Plant Production, Serra Talhada Academic Unit, Federal Rural University of Pernambuco, Av. Gregório Ferraz Nogueira, s/n, Serra Talhada 56909-535, PE, Brazil; 2 Department of Biodiversity, Institute of Biosciences, São Paulo State University, Rio Claro 13506-900, SP, Brazil; 3 Postgraduate Program in Agronomy, Federal University of Paraiba, Highway PB-079, Areia 58397-000, PB, Brazil

**Keywords:** IPA-10, IPA-11, algae extract, *Allium cepa* L., salinity

## Abstract

Salinity poses a significant challenge in modern agriculture, often inhibiting growth and yield, especially in sensitive crops like onions (*Allium cepa* L.). This study evaluated the effectiveness of a freshwater-algae-based biostimulant on two onion cultivars, Franciscana IPA-10 and Vale Ouro IPA-11, to mitigate saline irrigation’s adverse effects. Five biostimulant concentrations (0, 1, 2, 3, and 4 mL L^−1^, applied to the soil) were tested, along with two foliar treatments at 2 mL L^−1^ as controls. Our findings showed that applying 4 mL L^−1^ to the soil boosted growth rates to 1.0 cm per day (1), increased the potassium-to-sodium ratio in bulbs, and improved both average bulb weight by 25.11% and overall productivity by 24.28%, relative to untreated conditions. These results suggest that the biostimulant at 4 mL L^−1^ is an effective method to enhance resilience to saline stress and increase productivity in the IPA-10 and IPA-11 cultivars. However, while the biostimulant improved plant performance, it did not counteract the accumulation of salts in the soil. Therefore, additional management practices such as leaching and drainage are recommended to ensure sustainable onion production under saline water irrigation.

## 1. Introduction

Modern agriculture faces various challenges related to environmental factors, with salinity being one of the main constraints on agricultural production, especially in arid and semi-arid regions [1,2]. To mitigate these problems, biofertilizers have gained prominence as organic products that improve soil health and enhance nutrient absorption efficiency, promoting sustainable crop growth [3,4].

Excessive salinity negatively affects plant growth and physiology, particularly in sensitive crops like onion (*Allium cepa* L.) [5]. Exposure to high salt levels can reduce nutrient uptake, alter metabolism, and lead to severe declines in productivity [6,7]. In semi-arid regions of Brazil, such as the Caatinga biome, soil and water salinization is a critical factor limiting agricultural production [8].

Onion, one of the most widely cultivated vegetables globally, is notable for its nutritional and medicinal properties [9]. According to the Food and Agriculture Organization (FAO), Brazil ranked 13th among the largest global producers in 2022, with a production of 1.65 million tons, and the northeast region accounted for 20.6% of national production [10,11]. However, this crop is particularly sensitive to salinity, with an estimated reduction of 18.52% in productivity for each additional unit of electrical conductivity (EC) in the soil [12]. Freshwater-algae-based biostimulants have emerged as a promising solution for sustainable agricultural intensification [13]. Composed of phytohormones, polysaccharides, and amino acids, these products can enhance plant tolerance to abiotic stresses, such as salinity, while optimizing nutrient absorption [14,15].

Recent research has highlighted the beneficial effects of algae extracts, particularly from 121 species of the genera *Chlorella*, *Sargassum*, *Laminaria*, *Durvillaea*, *Ascophyllum*, and *Ecklonia* [16,17,18], on the growth of various crops, including tomato (*Solanum lycopersicum* L), papaya (*Carica papaya* L.)watermelon (*Citrullus lanatus*), spinach (*Spinacia oleracea*), rice (*Oryza sativa* L.), corn (*Zea mays* L.), and lettuce (*Lactuca sativa* L.) [19,20,21,22,23,24]. These biostimulants promote root development, leaf expansion, and bulb quality, as well as improve the physical and chemical parameters of the soil, increasing the water and nutritional efficiency of the plants [20,25].

Although previous research has indicated that algae contain bioactive compounds beneficial for plant growth, the application of these products in onions and their response to soil salinization, or even the behavior of the crop when irrigated with saline water, has yet to be fully investigated. Therefore, this study aims to evaluate the efficiency of freshwater-algae-based biostimulants in two onion cultivars (*Allium cepa* L.) under irrigation with saline water, seeking to mitigate the negative effects of salinity on growth, productivity, and bulb quality.

## 2. Results

### 2.1. Growth Parameters

**Figure 1:**
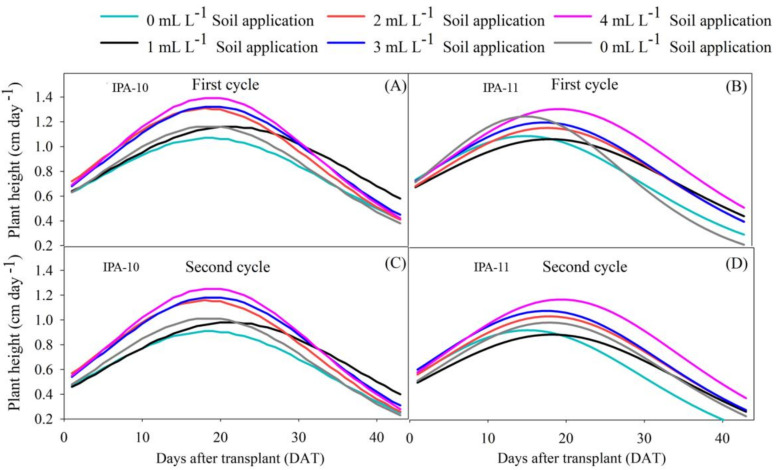
Growth rates of the aerial part of the plant (cm per day) during the vegetative phase of onion cultivars IPA-10 (**A**,**C**) and IPA-11 (**B**,**D**), subjected to irrigation with saline water under different doses and methods of biostimulant application.

The onion cultivars IPA-10 and IPA-11 exhibited a similar growth pattern, initially slow (until plant stand stabilization in the field (10 DAT)), reaching a growth rate of 1.0 cm per day (1) during the peak efficiency period (between 15 and 25 DAT) with the application of a dose of 4 mL L^−1^. At 30 DAT, there was a significant average decrease in the growth rate of the plants during the first cycle, amounting to 20.20% for IPA-10 and 29.49% for IPA-11 (Figure 1A,B). This result was replicated in the second cycle, with decreases of 23.70% and 29.70%, respectively, compared to the other applied doses (Figure 1C,D).

**Figure 2:**
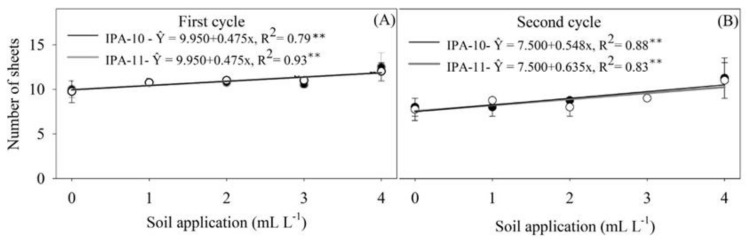
Number of leaves produced by the onion plants IPA-10 and IPA-11 in the first cycle (**A**) and in the second cycle (**B**) under saline irrigation with different doses of biostimulant applied to the soil. The bars represent the mean and the standard deviation of the observed values. ** Significant at *p* ≤ 0.01.

In Figure 2A,B, a similar pattern is observed between the first and second cycles regarding the number of leaves produced during the vegetative phase of cultivar IPA-11.

In both cycles, the dose of 4 mL L^−1^ resulted in the highest average number of leaves, surpassing the other treatments applied to the soil. For cultivar IPA-10, in the first cycle, only the dose of 0 mL L^−1^ showed a lower average number of leaves compared to the other doses. In the second cycle, the doses of 0 and 1 mL L^−1^ also resulted in lower values compared to the other doses.

**Figure 3:**
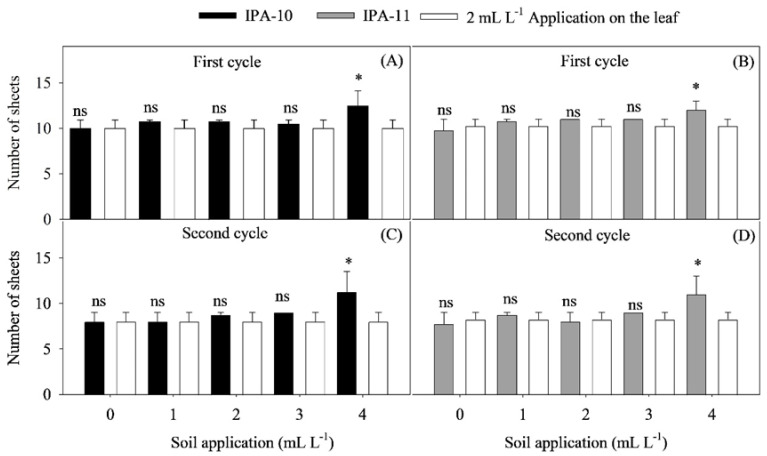
Comparison of the effect between the control dose (2 mL L^−1^ application on the leaf) and the different doses administered via soil application, considering the onion cultivars IPA-10 (**A**,**C**) and IPA-11 (**B**,**D**) during the first and second cycles, subjected to saline irrigation with different doses of biostimulant. Bars accompanied by (*) indicate significant differences compared to the control, while lines with (-) represent the standard deviation. Bars followed by (ns) indicate no significant differences compared to the control, according to the Dunnett test at a 5% probability level.

In Figure 3A–D, a similar pattern can be observed between cultivars IPA-10 and IPA-11 regarding the number of leaves produced during the vegetative phase, both in the first and second cycles. In both cultivars, the dose of 4 mL L^−1^ resulted in the highest average number of leaves. For the IPA-10 cultivar, there was an increase of 20% in the first cycle and 28.88% in the second cycle compared to the control treatment (2 mL L^−1^ application on the leaf) (Figure 3A,C). For the IPA-11 cultivar, the increases were 14.58% in the first cycle and 25% in the second cycle, relative to the control treatment (Figure 3B,D).

### 2.2. Yield Parameters

**Figure 4:**
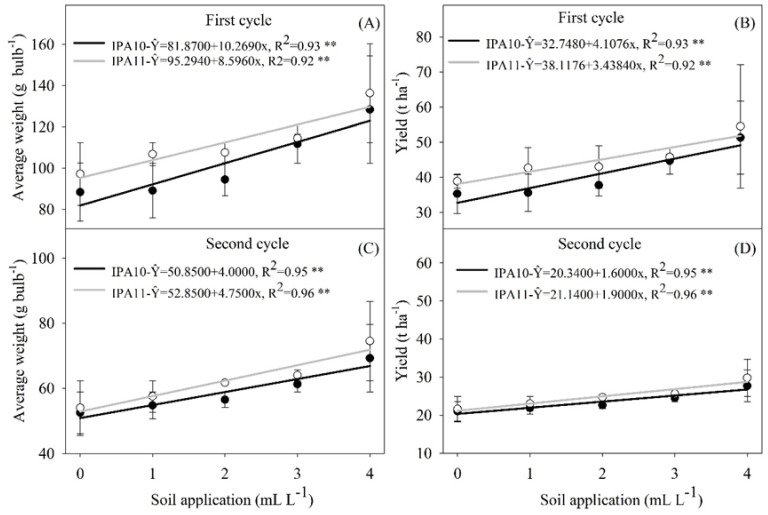
Average bulb weight (g bulb^−1^) (**A**,**C**) and total productivity (t ha^−1^) (**B**,**D**) of the onion varieties IPA-10 and IPA-11 subjected to saline irrigation with different doses and methods of biostimulant application. The bars represent the mean and the standard deviation of the observed values. ** Significant at *p* ≤ 0.01.

No interaction was observed between cultivars IPA-10 and IPA-11 and the different doses of biostimulant regarding the average bulb weight and total productivity (Figure 4A–D).

The doses of biostimulant applied to the soil had a significant impact on both the average bulb weight and the total productivity in both cultivars. In the IPA-10 cultivar, the 4 mL L^−1^ dose resulted in an increase of 12.93 to 31.71% in the average bulb weight and total productivity compared to the other soil-applied doses during the first cycle (Figure 4A,B). In the second cycle, the application of the same biostimulant dose (4 mL L^−1^) resulted in an increase of 28.70 to 40.07% in the average bulb weight and total productivity compared to the other soil-applied doses (Figure 4C,D).

The 3 and 4 mL L^−1^ doses of biostimulant resulted in a significant increase (9.27 and 23.81%, respectively) in the average bulb weight and total productivity compared to the 0, 1, and 2 mL L^−1^ doses applied to the soil during the first cycle of the IPA-11 cultivar (Figure 4A,B). In the second cycle, the 4 mL L^−1^ dose resulted in a significant increase (20.40%) in the average bulb weight and total productivity compared to the other doses applied to the soil (Figure 4C,D).

The application of the 4 mL L^−1^ biostimulant dose to the soil resulted in an average bulb weight for IPA-10 of 128.36 g bulb^−1^ in the first cycle, decreasing to 69.25 g bulb^−1^ in the second cycle. This same dose resulted in similar yields for the IPA-11 cultivar, with average bulb weights of 134.4 g bulb^−1^ in the first cycle and 74.50 g bulb^−1^ in the second cycle.

These results project an estimated productivity of 51.34 t ha^−1^ for the IPA-10 variety and 54.53 t ha^−1^ for the IPA-11 cultivar during the first cycle. In the second cycle, the projected productivity was 27.70 t ha^−1^ for IPA-10 and 28.80 t ha^−1^ for the IPA-11 cultivar. Compared to the first cycle, there was a reduction in productivity of 46.04% for IPA-10 and 47.18% for the IPA-11 cultivar in the second cycle.

**Figure 5:**
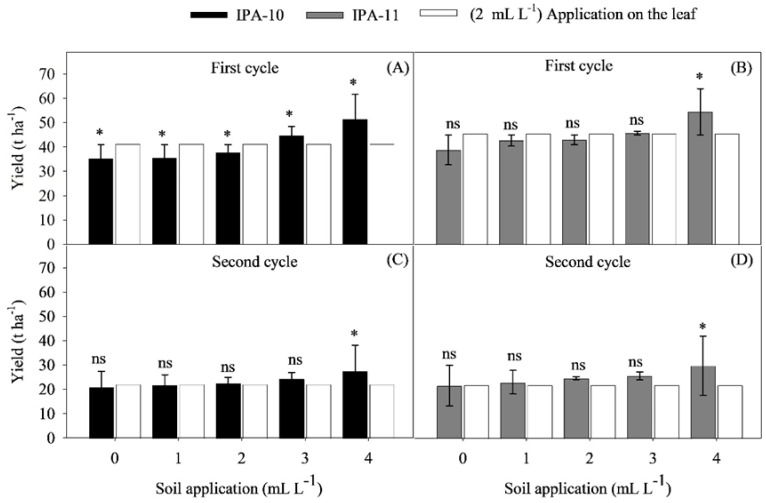
Comparison of the effect between the control dose (2 mL L^−1^, application on the leaf) and the different doses administered via soil application, considering the onion cultivars IPA-10 (**A**,**C**) and IPA-11 (**B**,**D**), subjected to irrigation with saline water at different doses of biostimulant. The bars marked with (*) indicate significant differences compared to the control, while the lines with (-) represent the standard deviation. The bars followed by (ns) indicate the absence of significant differences compared to the control, according to Dunnett’s test at a 5% probability level.

When comparing the treatments applied to the soil with the control, it was found that the application of the 0, 1, and 2 mL L^−1^ doses to the soil resulted in a total productivity that was lower (12.11%) than that of the control dose, except for the 3 and 4 mL L^−1^ doses, which resulted in a total productivity that was higher (16.52%) for the IPA-10 cultivar during the first cycle (Figure 5A).

In the second cycle, only the 4 mL L^−1^ dose applied to the soil resulted in a higher yield (20.48%) compared to the control dose for cultivar IPA-10 (Figure 5C). In both cycles of cultivar IPA-11, only the 4 mL L^−1^ dose applied to the soil resulted in a higher yield compared to the control dose (24.77 and 36.0 7%, respectively).

It was found that the 4 mL L^−1^ dose also led to substantial increases in the total yield of bulbs in the first and second cycles, recording increases of 105.36 and 10.80% for IPA-10, and 118.12 and 19.20% for IPA-11, respectively, compared to the estimated productivity forecast by the Agronomic Institute of Pernambuco (IPA) (25,000 Kg ha^−1^) under normal cultivation conditions.

**Figure 6:**
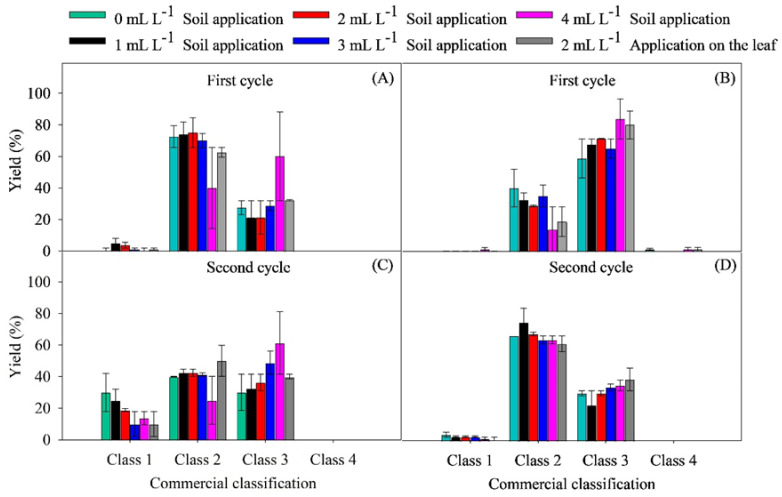
Commercial yield of onion varieties IPA-10 (**A**,**C**) and IPA-11 (**B**,**D**) subjected to irrigation with saline water under different doses and methods of biostimulant application. The bars represent the mean and the standard deviation of the observed values.

In the assessment of commercial yield (Figure 6A–D), the classification of bulbs according to size class is another indicator of the production quality achieved.

It is noticeable that the dosage of 4 mL L^−1^, applied to the soil, resulted in a greater quantity of commercial bulbs included in Class 3 for cultivar IPA-10 during the first (60.0%) and second cycles (61.25%), compared to the other doses (Figure 6A,C).

For cultivar IPA-11, in both cycles, the control dose of 2 mL L^−1^ and the dose of 4 mL L^−1^, both applied to the soil, resulted in a higher number of commercial bulbs that fell into Class 3, both in the first cycle (80.02 and 83.7%) and in the second (35.0 and 38.7%), compared to the other doses (Figure 6B,D). Regardless of the applied biostimulant dose, the classification of onion bulbs in the commercial Class 1 category was low for both cultivars.

### 2.3. Physical–Chemical Characteristics (Bulb Firmness, Titratable Acidity, Soluble Solids, and pH)

No statistically significant changes (*p* > 0.05) were identified for the bulb firmness, soluble solids content, titratable acidity, or pH of the onion cultivars IPA-10 and IPA-11 (Table 1). In other words, the applied treatments did not promote significant changes in the physical–chemical characteristics of the bulbs of the onion cultivars IPA-10 and IPA-11.

### 2.4. Evaluation of Potassium and Sodium Ions

**Figure 7:**
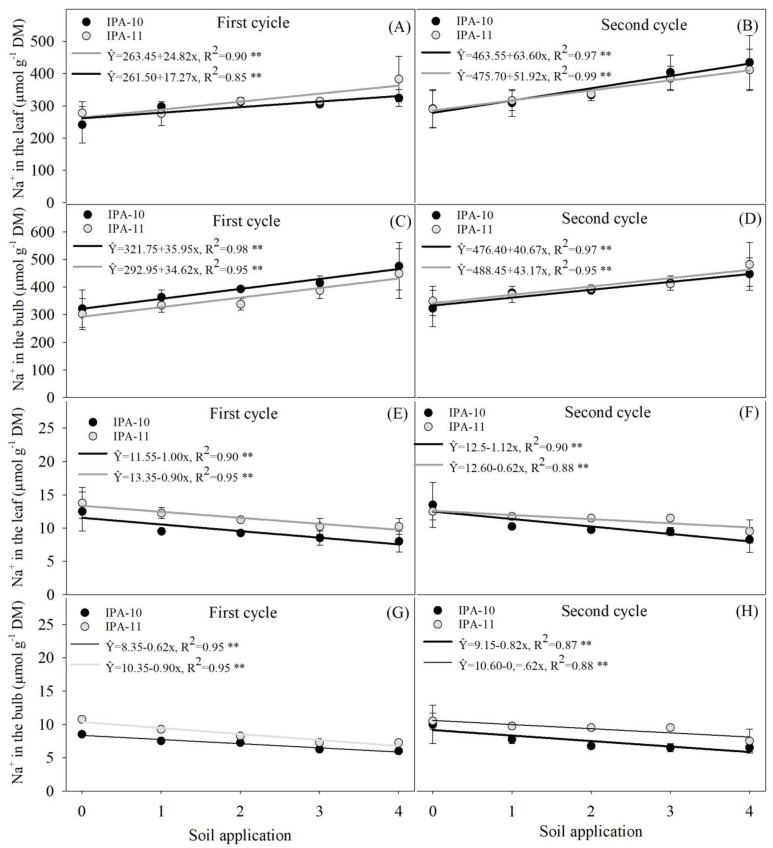
Potassium (K^+^) content in the leaves during the first cycle (**A**,**B**), potassium (K^+^) content in the bulbs during the first cycle (**C**,**D**), sodium (Na^+^) content in the leaves during the second cycle (**E**,**F**), and sodium (Na^+^) content in the bulbs during the second cycle (**G**,**H**). The bars represent the mean and the standard deviation of the observed values. ** Significant at *p* ≤ 0.01.

A positive response was observed in cultivar IPA-11 concerning the potassium (K^+^) content accumulated in the leaves with the application of 4 mL L^−1^ of the biostimulant, resulting in a significant increase in potassium (K^+^) content in the aerial part of the plant during the first cycle (27.27%) compared to the 0 mL L^−1^ dose (Figure 7A). On the other hand, cultivar IPA-10 did not show significant differences in the K^+^ levels present in the leaves across the different applied doses (Figure 7A).

In terms of the potassium (K^+^) levels in the bulbs of both cultivars, the application of 4 mL L^−1^ resulted in a significant increase in the absorption of this element during the first cycle, for both IPA-10 (32.36%) and IPA-11 (32.63%), compared to no application (0 mL L^−1^) (Figure 7C). During the second cycle, no significant differences were observed between the averages of the different biostimulant doses applied in terms of the potassium (K^+^) content accumulated in both the leaves and bulbs of onion plants, for both cultivars (Figure 7B,D). Additionally, no significant differences were found between the different doses applied and the sodium (Na^+^) content in both the leaves and bulbs of onion plants in both cultivars during the first cycle (Figure 7E).

These results remained consistent in the second cycle only for the IPA-11 cultivar (Figure 7F,H). However, in the IPA-10 cultivar, a significant decrease in sodium (Na^+^) accumulation in the bulbs was observed as the biostimulant doses increased. Specifically, without application (0 mL L^−1^), the sodium levels in the bulbs increased by approximately 35% compared to those found in the bulbs of this cultivar with the application of 4 mL L^−1^ (Figure 7E).

**Figure 8:**
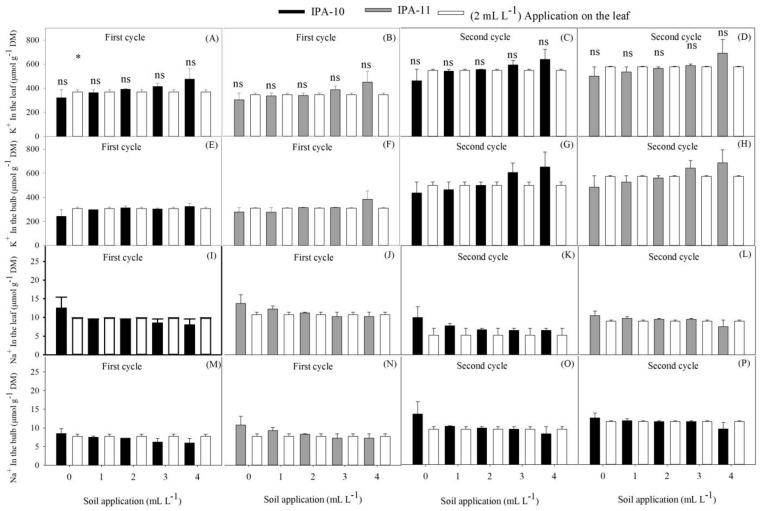
Comparison of the effect between the control dose (2 mL L^−1^ application on the leaf) and the different doses administered via soil application regarding potassium content in the leaves (K^+^) (**A**–**D**) and in the bulbs (**D**–**H**); and sodium in the leaves (Na^+^) (**F**,**I**–**L**) and in the bulbs (**M**–**P**) of the onion cultivars IPA-10 and IPA-11 during the first and second cycles. Bars marked with (*) indicate significant differences compared to the control, while lines with (-) represent standard deviation. Bars followed by (ns) indicate no significant differences compared to the control, according to Dunnett’s test at 5% probability.

When comparing the treatments applied to the soil with the control, no significant differences were observed in the potassium (K^+^) concentration in the leaves of both cultivars during the first and second cycles (Figure 8A–D). However, the concentration of K^+^ in the bulbs only increased for IPA-11 with the application of 4 mL L^−1^ compared to the control during the first cycle (22.44%) (Figure 8F). In the second cycle, there was no significant difference between the doses applied to the soil and the control (Figure 8G,H).

Regarding the sodium (Na^+^) content in the leaves and bulbs of both cultivars, no significant differences were observed between the doses applied to the soil compared to the control (Figure 8I–P).

**Figure 9:**
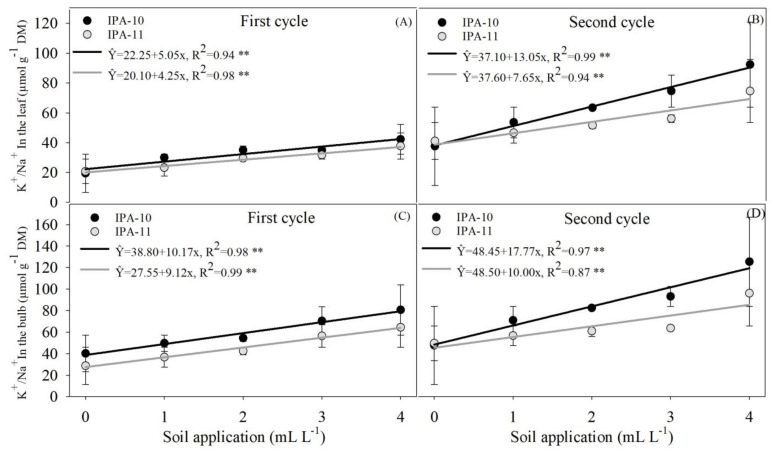
K^+^/Na^+^ ratio in the leaves during the first and second cycles (**A**,**B**) and K^+^/Na^+^ ratio in the bulbs during the first and second cycles (**C**,**D**). The bars represent the mean and standard deviation of the observed values. ** Significant at *p* ≤ 0.01.

The interaction between factors for the K^+^/Na^+^ ratio in the leaves was significant only for the IPA-11 cultivar during the first cycle, resulting in an increase of 45.03% compared to the 0 mL L^−1^ dose (Figure 9A). However, during the second cycle, a significant increase in the K^+^/Na^+^ ratio was observed with the application of the 4 mL L^−1^ dose, compared to the control, for both cultivars (61.67% for IPA-10 and 45.57% for IPA-11), relative to the 0 mL L^−1^ dose (Figure 9B).

The results for the K^+^/Na^+^ ratio in the bulbs showed a significant increase with the application of 4 mL L^−1^ of the biostimulant compared to the control treatment during the first cycle for both cultivars (50.15% for IPA-10 and 55.42% for IPA-11) in relation to the 0 mL L^−1^ dose (Figure 9C). However, the K^+^/Na^+^ ratio in the bulbs was significant only for the IPA-11 cultivar during the second cycle, with a 48.31% increase compared to the 0 mL L^−1^ dose (Figure 9D).

**Figure 10:**
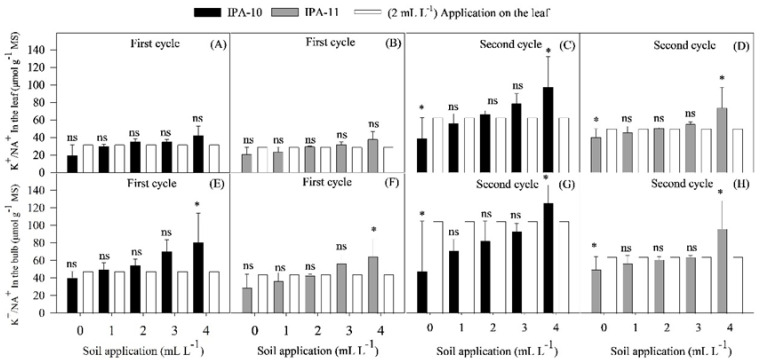
Comparison of the effect between the control dose (2 mL L^−1^ application on the leaf) and the various doses administered via soil application regarding the K^+^/Na^+^ ratio in the leaves during the first (**A**,**B**) and second cycles (**C**,**D**) and the K^+^/Na^+^ ratio in the bulbs during the first (**E**,**F**) and second cycles (**G**,**H**). Bars marked with (*) indicate significant differences compared to the control, while lines with (-) represent the standard deviation. Bars followed by (ns) indicate the absence of significant differences compared to the control, according to the Dunnett test at 5% probability.

When comparing the treatments applied to the soil and the control, no significant differences were observed in the K^+^/Na^+^ ratio in the leaves of both cultivars during the first cycle (Figure 10A,B).

In the second cycle, a significant decrease in the K^+^/Na^+^ ratio was observed with the 0 mL L^−1^ dose compared to the control (37.60% for IPA-10 and 19.71% for IPA-11). In contrast, there was a significant increase for both cultivars IPA-10 and IPA-11 with the 4 mL L^−1^ dose (47.59% and 32.21%, respectively) compared to the control (Figure 10C,D).

The analyzed data for the K^+^/Na^+^ ratio in the bulbs between the soil treatments and the control group suggested a significant increase for both cultivars during the first cycle with the application of the 4 mL L^−1^ dose compared to the control (41.20% for IPA-10 and 31.24% for IPA-11) (Figure 10E,F). However, during the second cycle, examining the K^+^/Na^+^ ratio in the bulbs in the soil treatments and in the foliar application (control) revealed a significant reduction for both cultivars (54.48 % for IPA-10 and 22.53% for IPA-11) when subjected to the 0 mL L^−1^ dose compared to the control, along with an increase with the application of the 4 mL L^−1^ dose compared to the control (16.41% for IPA-10 and 33.29% for IPA-11) (Figure 10G,H).

### 2.5. Photosynthetic Pigments

No significant differences (*p* > 0.05) were observed for chlorophyll a, chlorophyll b, total chlorophylls, or carotenoids in cultivars IPA-10 and IPA-11 (Table 2).

### 2.6. Electrical Conductivity of the Soil Saturation Extract

The data presented in Table 3 indicate that the electrical conductivity of the soil saturation extract changed from the first to the second cultivation cycle, showing an average increase of 76.47%.

The average increase in electrical conductivity of the soil saturation extract compared to the initially measured electrical conductivity at the beginning of the first cycle was 431%, while this increase reached 200% when comparing it with the EC measured at the beginning of the second productive cycle. This indicates that the applied doses of biostimulant did not have an effect on the progressive increase in soil electrical conductivity under permanent irrigation with saline water of Class C3 over a period of 8 months.

## 3. Discussion

**Figure 11:**
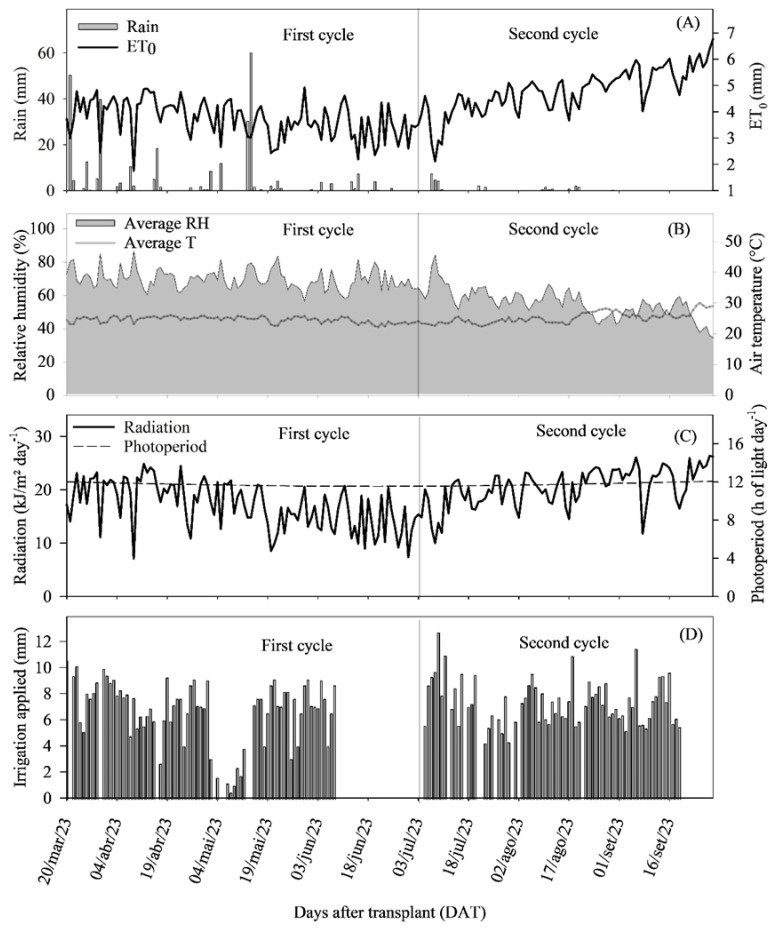
Observed climatic variables (**A**,**B**), radiation and photoperiod (**C**), and applied irrigation (**D**) throughout the two cultivation cycles of the onion cultivars IPA-10 and IPA-11. Serra Talhada, PE, 2023.

The results of this study show that the application of biostimulants significantly improved the development of onion plants (*Allium cepa* L.) under saline water irrigation, increasing growth and the number of leaves (Figure 1A–D, (Figure 2A,B and (Figure 3A–D). The two cultivation cycles, carried out under different climatic conditions (Figure 11A–D), demonstrated that, in the first cycle, the accumulated precipitation of 306.4 mm and the average relative humidity of 69.45% favored growth. The average temperature of 24.8 °C, a photoperiod of 11.72 h, and the average radiation of 17.78 kJ m^−2^ per day created ideal conditions, where irrigation salinity (1.68 dS m^−1^) did not severely impact the plants’ metabolism.

In the second cycle, the climatic conditions posed a substantial challenge, with only 29.8 mm of precipitation, 55% relative humidity, and slightly higher temperatures (25.3 °C). This scenario was aggravated by higher solar radiation (21.20 kJ m^−2^ per day) and the increased salinity accumulated by irrigation (5667 kg ha^−1^ of salts). These conditions, combined with high evapotranspiration demand, with peaks of up to 6.76 mm per day, created a highly unfavorable environment for onion cultivation, resulting in greater saline and water stress.

These factors, such as salinity and low humidity, negatively impact the growth of sensitive plants like onions by reducing water absorption and causing the accumulation of toxic ions in plant tissues [26,27]. However, the biostimulant Ferticell Universal 3-0-1^®^, composed of 25% freshwater microalgae extract and rich in essential nutrients (N, P, K, Zn, Mn, and B), proved effective in mitigating these adverse effects, as observed in the plants’ performance during the second cycle. The presence of cytokinins in the biostimulant contributed to promoting cell division and regulating water balance, enhancing tolerance to salt stress [28,29].

Furthermore, the higher solar radiation observed in the second cycle may have enhanced photosynthesis, a process that was possibly optimized by the action of the biostimulant. The bioactive compounds present in the biostimulant, such as polysaccharides and phytohormones, likely acted as physiological modulators, promoting an increase in photosynthetic activity and antioxidant synthesis, even under adverse conditions of salinity and low humidity [30]. These compounds are known to stimulate carbohydrate metabolism and improve plant resistance to abiotic stress, resulting in greater water use efficiency and nutrient assimilation.

The results of this study support previous findings on the growth-promoting effect of algae-based biostimulants, which increase nutritional efficiency and resilience to abiotic stress. The literature already highlights that algae contain chemical compounds that influence various plant growth processes, even at low concentrations [31,32,33,34]. These results are also consistent with those of previous studies, such as those by [20,35], which observed similar effects on the growth of onions and other vegetables, including tomatoes [36], eggplant [37], beetroot [38], and Swiss chard [39].

The nutritional composition of the biostimulant, which includes nitrogen, phosphorus, potassium, and micronutrients such as zinc, manganese, and boron, was decisive for the plants’ positive performance. Potassium (K^+^), in particular, plays a crucial role in osmotic regulation and ionic homeostasis, essential for salt stress tolerance [40]. The increase in leaf potassium content in plants treated with higher doses of the biostimulant indicates improved nutrient uptake and transport, favoring a more efficient K^+^/Na^+^ ratio. This balance is fundamental for maintaining cellular integrity and continuing physiological processes under saline conditions [41,42,43,44].

The presence of micronutrients such as zinc (Zn) and manganese (Mn) contributed to the activation of antioxidant enzymes, such as superoxide dismutase and catalase, which are crucial for detoxifying the reactive oxygen species (ROS) generated in plants under salt stress [45]. Studies indicate that algae-based biostimulants also contain phenolic compounds and polysaccharides, which increase the antioxidant capacity of plants [46], helping to reduce oxidative damage during periods of higher salinity, resulting in increased plant height and leaf number [47].

The application of the biostimulant in increasing doses resulted in significant improvements in the average bulb weight and yield per hectare, especially in the first cycle when climatic conditions were more favorable (Figure 4A,B,D; Figure 5A–D). The production of larger bulbs (≥50 mm) was directly proportional to the doses of the biostimulant, demonstrating a positive response (Figure 6A–D). This result indicates that the biostimulant not only supports the initial growth of the plants, but also promotes continued development during the critical growth phases of the bulbs, even under saline conditions.

This behavior is consistent with studies highlighting the ability of algae-based biostimulants to improve water and nutrient use efficiency in crops subjected to salt stress [39,48]. The production of larger bulbs is positively correlated with increased productivity and also contributes to greater profitability, as bulbs with a diameter of less than 50 mm generally have a lower market value compared to larger ones [49,50].

The ability of the biostimulant to mitigate the deleterious effects of salinity may be associated with the activation of hormonal pathways, particularly those linked to abscisic acid (ABA) and cytokinins, which regulate the response to salt stress [51]. The presence of cytokinins in the algal extract may have contributed to the increase in leaf number and leaf area, promoting greater carbon assimilation and, consequently, an increase in biomass production. In the study by [52], the application of freshwater algae resulted in an 18.3% increase in the yield of green onion and spinach compared to the untreated group, corroborating the results of the present research, where higher doses of the biostimulant led to a substantial increase in average bulb weight (Figure 6A–D).

Even with the increase in soil salinity (Table 3), plants treated with 4 mL L^−1^ of the biostimulant exhibited productivity superior to the average estimated by the Agronomic Institute of Pernambuco for the cultivars used. Previous studies have also highlighted that the use of biostimulants based on algal extracts resulted in increases in productivity and/or yield in various crops, including wheat, tomato, green onion, onion, spinach, rice, corn, watermelon, soybeans, and lettuce [19,20,21,22,23,24,33,35,53,54,55].

**Figure 12:**
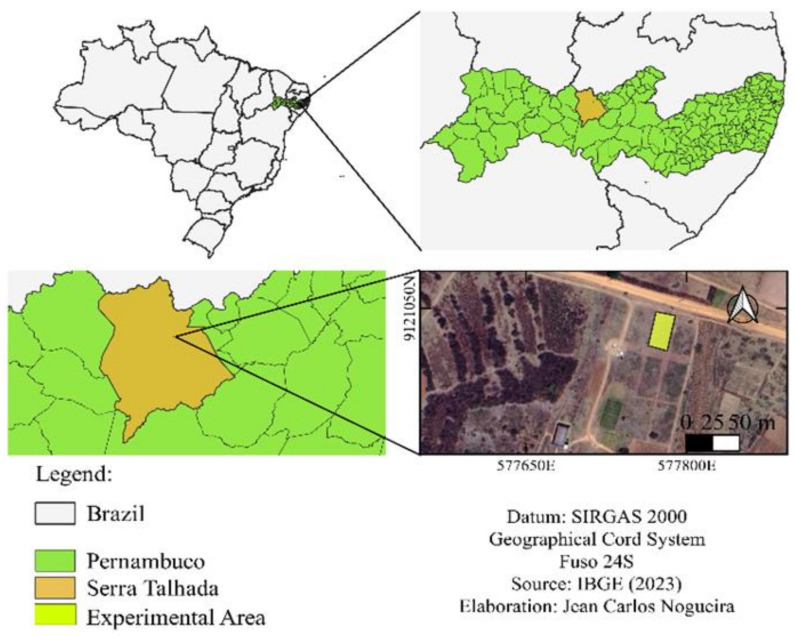
Location of the experimental area—UFRPE/UAST, Serra Talhada, PE, Brazil, December 2023 [11].

The performance of the plants treated with the biostimulant can be attributed to the greater absorption of nutrients, especially potassium, as evidenced by the elevated levels of this element in the leaves and bulbs (Figure 9A–D). This increase contributed to a more favorable K^+^/Na^+^ ratio under saline conditions (Figure 12). Potassium plays crucial roles in osmotic regulation, respiration, carbohydrate metabolism, protein synthesis, transport, photosynthesis, and water balance [56,57]. According to [58], the accumulation of potassium promotes the formation of an osmotic gradient that facilitates water movement, which is essential for regulating stomatal opening and maintaining cellular turgor.

The results indicated that the increase in irrigation salinity significantly raised the sodium (Na^+^) levels in the leaves of onion plants, especially in plots that did not receive the biostimulant. Plants treated with the highest doses of the biostimulant showed lower Na^+^ accumulation in the leaf tissues, suggesting that the biostimulant helped to reduce sodium absorption or its efficient compartmentalization, minimizing toxic effects (Figure 7E–H). This mechanism may be related to the increased activity of ion transport pumps that help to keep Na^+^ out of cells susceptible to its accumulation, as described in studies on the response to salt stress.

Potassium is an essential nutrient for maintaining cellular turgor and osmotic regulation, especially under salt stress, where its elevated presence is crucial for neutralizing the harmful effects of sodium (Na^+^). In this study, the concentration of potassium ions (K^+^) found in the biostimulant and potassium fertilization was significantly high, exceeding 1200 µmol L^−1^. This elevated concentration facilitates potassium absorption by the plant roots [59]. As a result, the K^+^/Na^+^ ratios increased with the doses of biostimulant applied under salt stress (Figure 9A–D).

The increase in the K^+^/Na^+^ ratio is beneficial, as it helps to improve stomatal conductance and maintains hormonal balance and photosynthesis during salt stress [60]. Treatments with other biostimulants have also shown a positive effect on the K^+^/Na^+^ ratio [61]. These factors are important for plant growth and development [62] and help to reduce the risk of physiological disorders [63].

When comparing treatments with different doses of biostimulant administered via soil application to the control applied via foliar application, it was observed that the higher doses of biostimulant in the soil resulted in significantly better performance. The foliar application of the control did not achieve the same efficiency in growth, yield, and mitigation of salinity effects, showing lower increases in K^+^ levels (Figure 10E–H) and a higher Na^+^/K^+^ ratio. This indicates that applying the biostimulant to the soil is more effective for promoting plant health under adverse salinity conditions.

This reinforces the idea that applying the biostimulant through the soil allows for more efficient absorption of nutrients and bioactive compounds, especially under saline conditions, where the soil acts as a reservoir, facilitating nutrient availability throughout the plant growth cycle. Similar results were found by [52], who reported increases in potassium and other mineral levels in chives and spinach plants treated with freshwater algae, highlighting the effectiveness of this approach for plant growth.

When used as soil additives, algal extracts can significantly contribute to plant nutrition by optimizing the physiological solutions associated with growth [34]. This improvement in nutrient absorption can be crucial for the healthy development of plants, particularly those under salt stress, where competition for nutrients becomes more intense. Therefore, the proper application of biostimulants can not only mitigate the negative effects of salinity, but also enhance the overall performance of crops.

## 4. Materials and Methods

### 4.1. Study Area

This experiment was conducted over two growing cycles in the experimental field of the Federal Rural University of Pernambuco—Academic Unit of Serra Talhada (UFRPE/UAST), in Serra Talhada, PE, Brazil (7°59′ S, 38°15′ W, altitude: 499 m) (Figure 12).

The climate of the region is hot semi-arid (BSwh’), with an average annual precipitation of 667.2 mm and potential evapotranspiration ranging from 1800 to 2000 mm. The average temperature is 26.5 °C, and the relative humidity is 62.7% [64].

### 4.2. Experimental Design

The experiment was conducted using a randomized complete block design with a factorial scheme of 5 × 2 + 2, with four repetitions. The first factor consisted of doses of biostimulant (0, 1, 2, 3, and 4 mL L^−1^) applied to the soil. The second factor involved the onion cultivars Franciscana IPA-10 (purple) and Vale Ouro IPA-11 (yellow), with two control treatments (IPA-10 and IPA-11), utilizing a foliar application of the biostimulant (2 mL L^−1^) (the dose recommended by the manufacturer). The experimental area totaled 180 m2, consisting of 48 plots of 1 m^2^ each, containing 80 plants per plot, with 20 central plants considered experimental units, aiming to minimize the influence of external factors that may affect the plants located at the edges of the plots.

### 4.3. Management of the Experimental Area

The applications of the biostimulant began eight days after transplanting and occurred weekly, with a total of eight applications per cycle. The biostimulant used was Ferticell Universal 3-0-1^®^ marketed by the company Agroplama, headquartered in Malaga, Spain, containing 25% unicellular freshwater algae extract, which is completely neutral and biologically active, in addition to 2.72% nitrogen (N), 2.57% phosphorus (P), 1.14% potassium (K), 0.52% sulfur (S), 0.15% zinc (Zn), 0.05% boron (B), 58.7 mg L^−1^ magnesium (Mg), 0.25 mg L^−1^ copper (Cu), and 150.03 mg L^−1^ manganese (Mn). The solution was applied to the soil between the planting rows via fertigation (1.5 L per plot) and via foliar application using a pressurized sprayer. The irrigation management was carried out by drip irrigation, controlled with FDR sensors at three depths (10, 20, and 30 cm), with daily monitoring of meteorological conditions.

### 4.4. Climatic and Soil Conditions

During the first cycle, the precipitation was 306.4 mm, with the highest volumes recorded between the 1° and 11° days and the 55° and 57° days after transplanting (DAT). In the second cycle, the precipitation was only 29.8 mm. The reference evapotranspiration (ET0) varied between 3.85 and 4.78 mm per day, with peaks of up to 6.76 mm per day (Figure 11A).

The average temperature was 24.8 °C in the first cycle and 25.3 °C in the second cycle, with average relative humidity of 69.45% and 55.0%, respectively (Figure 11B). The average photoperiod was 11.72 h of light per day in the first cycle and 11.82 h in the second cycle. The average radiation recorded during these periods was 17.78 kJ m^−2^ per day in the first cycle and 21.20 kJ m^−2^ per day in the second cycle (Figure 11C).

The soil in the experimental area is classified as typical Eutrophic Haplic Cambisol, with sandy loam texture. Before preparing the area, soil samples were collected for physical–chemical analysis (texture and fertility) (Table 4).

### 4.5. Cultivation, Irrigation, and Fertilization

Planting was carried out manually in two stages, as follows: 20 February 2023, for the first cycle, and 3 June 2023, for the second cycle. The seedlings were transplanted 30 days after emergence, with a spacing of 0.20 m between the plants and 0.10 m between double rows, resulting in a density of 400,000 plants per hectare. Mineral and organic fertilization was conducted based on soil analysis and the crop’s nutrient requirements. A total of 250 kg ha^−1^ of P_2_O_5_ (single superphosphate) was applied to the soil 60 days before transplanting, along with 66.67 kg ha^−1^ of N (urea) and 20 t ha^−1^ of goat manure. Nitrogen top-dressing was applied through fertigation in 14 applications, totaling 120 kg ha^−1^ of N. Additionally, 45 kg ha^−1^ of K_2_O, 35 kg ha^−1^ of S, and 2 kg ha^−1^ of B were applied in 8 applications. Urea, potassium chloride, magnesium sulfate, and boric acid were used as nutrient sources.

Irrigation was performed daily using a drip system, with a total irrigation volume of 491.26 mm in the first cycle and 527.10 mm in the second cycle (Figure 11D). Water samples used for irrigation were collected for physical and chemical analysis, and the water was classified as C3S1, with an electrical conductivity of 1.68 dS m^−1^, resulting in the addition of approximately 5282 kg ha^−1^ of salts in the first cycle and 5667 kg ha^−1^ in the second cycle (Table 5).

### 4.6. Data Collected

The growth of the onion plants was monitored from the central rows to avoid any influences from the edges. In each experimental plot, five plants were randomly selected and marked in the central rows. These plants were followed throughout the growth period for 46 days after transplanting (DAT), which corresponds to the vegetative phase of the crop, characterized by rapid leaf growth, where new leaves continue to be produced until the onset of bulb formation. The plant height and the number of leaves were recorded during this period as growth parameters.

The harvest of the bulbs from the first cycle took place on 16 June 2023, and the harvest of the second cycle was conducted on 29 September 2023, when 90% of the plants were at a physiologically mature stage (90 DAT), characterized by leaf decline. For bulb collection, 20 plants were randomly selected from the four central rows, avoiding the edges of the plots. The bulbs were then subjected to a curing process in the laboratory at an ambient temperature ranging between 25 and 30 °C for 10 days. After curing, the bulbs were weighed and measured for the characterization of yield components and quality.

#### 4.6.1. Growth Parameters

Data regarding plant height were collected during five biometric campaigns using measuring tape over a period of 43 days after transplanting (DAT), corresponding to the “adult or plant” phase, characterized by relatively rapid leaf growth. During this 43 DAT period, new leaves continued to be produced until the onset of bulb formation. Once the bulb growth phase began, leaf growth was halted. At the end of the 43 DAT period, the number of leaves produced by the plants was counted manually.

#### 4.6.2. Yield Parameters

The average bulb weight (in grams), total bulb production (in kg per hectare), and bulb production according to the minimum quality standards established by the Brazilian Ministry of Agriculture, Livestock, and Supply [65] were analyzed. The bulbs were classified based on the following criteria: Class 1—bulbs with a diameter of less than 35 mm and unfit for commercialization (non-marketable); Class 2—bulbs with a diameter between 35 and 50 mm; Class 3—bulbs with a diameter between 51 and 70 mm; and Class 4—bulbs with a diameter between 71 and 90 mm.

#### 4.6.3. Physical–Chemical Characteristics of the Bulbs

The firmness of the bulbs was measured in Newtons (N) using a bench-mounted penetrometer, model IMPAC 100 Kg IP-90COM^®^, with a tip diameter of 10 mm and a penetration depth of 7 mm. The readings were taken at the middle equatorial portion of three randomly selected bulbs at two equidistant points on opposite sides, after the removal of the bulbs’ dry skin.

To determine the titratable acidity, soluble solids content, and pH, the onion bulbs were blended in a blender until a juice was formed. The titratable acidity was measured according to the method described by [66], using a 1 mL aliquot of the bulb juice dissolved in 10 mL of pure demineralized water, to which three drops of 1% phenolphthalein were added. The titration was then performed until the endpoint was reached using a standardized NaOH solution (0.1 N).

The soluble solids content was directly determined using approximately 1 mL of the bulb juice, with a digital refractometer DBR45 (refractive index range of 1.3330–1.4098) that had automatic temperature compensation. The pH was directly measured using 10 mL of the bulb juice with the aid of a bench potentiometer, model R-TEC-7/2-MP^®^.

#### 4.6.4. Evaluation of Chloride, Potassium, and Sodium Ions in Plants

To obtain the quantities of chloride, potassium, and sodium ions, two onion plants were collected and dried in an oven at 60 °C until reaching a constant weight. Subsequently, samples of the leaves and bulbs were separated and ground in a knife mill. Samples of 50 mg of the dry material were collected to determine the chloride content in the aerial parts and bulbs, following the method of Malavolta et al. (1997) [67]. This method uses potassium chromate as an indicator solution and silver nitrate for titration.

The determination of sodium and potassium levels was also carried out according to the methodology of [67]. A sample of 50 mg of the dry material was incubated in 10 mL of ultrapure water and boiled in a water bath at 100 °C for 1 h. After this process, the extracts were filtered through filter paper and subjected to flame photometry to determine the levels of Na^+^ and K^+^. These levels were estimated based on standard curves for each element (NaCl and KCl), with values ranging from 0 to 1000 µM.

#### 4.6.5. Photosynthetic Pigments in the Leaves

The analyses were conducted using leaf samples collected during the early bulb formation stage. The samples were collected directly in the field, immediately placed in a refrigerated box, and transported to the Plant Analysis Laboratory. Five plants were collected from each experimental unit, selecting the tallest leaf at 47 days after transplanting (DAT). In the laboratory, 0.2 g of fresh plant material was extracted. The extraction was performed in test tubes containing 10 mL of 95% ethanol, and the samples were kept at 4 °C for 48 h.

After extraction, the concentrations of chlorophyll a (Chl a), chlorophyll b (Chl b), total chlorophyll (Chl a+b), and carotenoids (Car) were estimated according to the method of [68] using a spectrophotometer to measure absorbance values at 470, 648, and 664 nm.

### 4.7. Data Treatment and Statistical Analysis

The data were subjected to normality analysis using the Shapiro–Wilk test at a 5% significance level, and homogeneity was also assessed at a 5% significance level. Subsequently, an analysis of variance (ANOVA) was performed using the F-test (*p* < 0.05). When statistical significance was observed, the means were compared using Tukey’s test at a 5% probability level. Additionally, regression analysis was conducted when an effect of the biostimulant doses was identified.

The criteria for model selection included biological parameters, the significance of the estimated regression coefficients, and the values of the coefficient of determination (R^2^). To compare the control treatment (dose applied via foliar application) with different doses applied through the soil, Dunnett’s test was used (*p* < 0.05). To analyze the data over time, four-parameter sigmoid mathematical models were applied. All statistical analyses were conducted using R software, version 4.4.1 [69], while SigmaPlot version 13.0 [70] was used for fitting regression curves and creating graphs.

## 5. Conclusions

The action of the freshwater-algal-extract-based biostimulant favored the adaptation of onion cultivars to the stress conditions generated by irrigation with saline water, resulting in significant improvements in growth, productivity, and commercial yield of the crop. The use of the biostimulant at a concentration of 4 mL L^−1^ proved effective in facilitating potassium (K^+^) absorption by the plants, promoting an increase in the K^+^/Na^+^ ratio, which positively reflected on the productivity and commercial yield of the onion varieties.

Although the application of the biostimulant brought benefits, it was unable to reduce the impact of saline water on the secondary salinity of the soil. Therefore, the application of leaching fractions and the use of agricultural drainage remain necessary for strict control of salinity in the soil profile. While the research results are promising and aligned with the existing literature on the use of biostimulants in agriculture, it is essential to consider the specific characteristics of the crop or variety to be cultivated, as well as the cultivation conditions, when opting to use these products. Finally, further research is recommended to gain a deeper understanding of the underlying mechanisms and to improve the application of biostimulants in different crops and environments.

## Figures and Tables

**Table 1:** Physico-chemical variables of onion cultivars IPA-10 and IPA-11 irrigated with saline water under different doses of biostimulant.

**First Cycle**								
	**Firmness (N)**		**SS (Brix°)**		**TA (%)**		**pH**	
**Doses (mL L^−1^)**	**IPA-10**	**IPA-11**	**IPA-10**	**IPA-11**	**IPA-10**	**IPA-11**	**IPA-10**	**IPA-11**
0—Soil application	60 ^ns^	65 ^ns^	10.3 ^ns^	9.6 ^ns^	0.66 ^ns^	0.65 ^ns^	5.38 ^ns^	5.50 ^ns^
1—Soil application	61 ^ns^	61 ^ns^	10.3 ^ns^	9.9 ^ns^	0.65 ^ns^	0.65 ^ns^	5.28 ^ns^	5.39 ^ns^
2—Soil application	50 ^ns^	63 ^ns^	10.0 ^ns^	9.9 ^ns^	0.66 ^ns^	0.65 ^ns^	5.42 ^ns^	5.32 ^ns^
3—Soil application	62 ^ns^	63 ^ns^	10.3 ^ns^	9.7 ^ns^	0.66 ^ns^	0.65 ^ns^	5.33 ^ns^	5.35 ^ns^
4—Soil application	63 ^ns^	69 ^ns^	10.1 ^ns^	9.6 ^ns^	0.67 ^ns^	0.66 ^ns^	5.32 ^ns^	5.36 ^ns^
2—Application on the leaf	62 ^ns^	62 ^ns^	9.25 ^ns^	10.0 ^ns^	0.65 ^ns^	0.68 ^ns^	5.68 ^ns^	5.68 ^ns^
*p*-value	0.20	0.33	0.32	0.56	0.25	0.11	0.20	0.41
**Second Cycle**								
	**Firmness (N)**		**SS (Brix°)**		**AT (%)**		**pH**	
**Doses (mL L^−1^)**	**IPA-10**	**IPA-11**	**IPA-10**	**IPA-11**	**IPA-10**	**IPA-11**	**IPA-10**	**IPA-11**
0—Soil application	52 ^ns^	57 ^ns^	9.0 ^ns^	9.2 ^ns^	0.56 ^ns^	0.55 ^ns^	5.34 ^ns^	5.42 ^ns^
1—Soil application	52 ^ns^	57 ^ns^	8.5 ^ns^	9.2 ^ns^	0.55 ^ns^	0.53 ^ns^	5.24 ^ns^	5.31 ^ns^
2—Soil application	50 ^ns^	52 ^ns^	8.9 ^ns^	9.0 ^ns^	0.56 ^ns^	0.54 ^ns^	5.38 ^ns^	5.24 ^ns^
3—Soil application	42 ^ns^	55 ^ns^	8.8 ^ns^	9.1 ^ns^	0.54 ^ns^	0.54 ^ns^	5.29 ^ns^	5.27 ^ns^
4—Soil application	54 ^ns^	69 ^ns^	9.9 ^ns^	9.1 ^ns^	0.53 ^ns^	0.54 ^ns^	5.26 ^ns^	5.28 ^ns^
2—Application on the leaf	55 ^ns^	60 ^ns^	9.0 ^ns^	9.1 ^ns^	0.53 ^ns^	0.54 ^ns^	5.28 ^ns^	5.25 ^ns^
*p*-value	0.20	0.33	0.54	0.25	0.59	0.51	0.63	0.73

*p*-value indicates the probability of observing a difference as large or larger than the one observed under the null hypothesis. The means followed by “ns” indicate that there are no significant differences concerning the doses and the control (2 mL L^−1^ application on the leaf), based on the Dunnett (*p* < 0.05) and Tukey (*p* < 0.05) tests, between the doses applied to the soil and the control dose (2 mL L^−1^ application on the leaf). N = Newton; SS = soluble solids; AT = titratable acidity; and pH = hydrogen potential.

**Table 2:** Photosynthetic pigments of onion cultivars IPA-10 and IPA-11 irrigated with saline water under different biostimulant doses.

**First Cycle**								
	**Chlorophyll *a*** **(mg g^−1^)**		**Chlorophyll *b*** **(mg g^−1^)**		**Total Chlorophyll (mg g^−1^)**		**Carotenoids** **(mg g^−1^)**	
**Doses (mlL^−1^)**	**IPA-10**	**IPA-11**	**IPA-10**	**IPA-11**	**IPA-10**	**IPA-11**	**IPA-10**	**IPA-11**
0—Soil application	8.74 ^ns^	9.10 ^ns^	3.99 ^ns^	5.02 ^ns^	12.73 ^ns^	14.12 ^ns^	2.27 ^ns^	1.13 ^ns^
1—Soil application	8.74 ^ns^	8.34 ^ns^	3.67 ^ns^	4.08 ^ns^	12.41 ^ns^	12.42 ^ns^	1.98 ^ns^	1.77 ^ns^
2—Soil application	11.55 ^ns^	12.07 ^ns^	5.01 ^ns^	5.52 ^ns^	16.56 ^ns^	17.60 ^ns^	2.80 ^ns^	2.42 ^ns^
3—Soil application	11.72 ^ns^	13.85 ^ns^	4.80 ^ns^	5.56 ^ns^	16.53 ^ns^	19.41 ^ns^	2.57 ^ns^	3.60 ^ns^
4—Soil application	12.87 ^ns^	11.39 ^ns^	5.45 ^ns^	5.06 ^ns^	18.33 ^ns^	16.45 ^ns^	2.80 ^ns^	2.58 ^ns^
2—Application on the leaf	11.55 ^ns^	11.63 ^ns^	5.05 ^ns^	5.65 ^ns^	16.61 ^ns^	17.29 ^ns^	2.50 ^ns^	2.11 ^ns^
*p*-value	0.44	0.45	0.83	0.15	0.52	0.29	0.99	0.55
**Second Cycle**								
	**Chlorophyll *a*** **(mg g^−1^)**		**Chlorophyll *b*** **(mg g^−1^)**		**Total Chlorophyll (mg g^−1^)**		**Carotenoids** **(mg g^−1^)**	
**Doses (mlL^−1^)**	**IPA-10**	**IPA-11**	**IPA-10**	**IPA-11**	**IPA-10**	**IPA-11**	**IPA-10**	**IPA-11**
0—Soil application	8.42 ^ns^	6.48 ^ns^	3.12 ^ns^	3.02 ^ns^	11.54 ^ns^	9.05 ^ns^	2.08 ^ns^	1.53 ^ns^
1—Soil application	8.05 ^ns^	6.51 ^ns^	3.10 ^ns^	4.73 ^ns^	11.15 ^ns^	11.24 ^ns^	1.99 ^ns^	1.54 ^ns^
2—Soil application	8.19 ^ns^	6.94 ^ns^	3.75 ^ns^	3.32 ^ns^	12.94 ^ns^	10.24 ^ns^	2.18 ^ns^	1.63 ^ns^
3—Soil application	9.04 ^ns^	8.07 ^ns^	3.68 ^ns^	3.48 ^ns^	12.72 ^ns^	11.55 ^ns^	2.21 ^ns^	1.87 ^ns^
4—Soil application	9.53 ^ns^	8.29 ^ns^	4.19 ^ns^	3.49 ^ns^	13.72 ^ns^	11.78 ^ns^	2.21 ^ns^	1.87 ^ns^
2—Application on the leaf	8.94 ^ns^	6.06 ^ns^	3.61 ^ns^	3.15 ^ns^	12.55 ^ns^	9.21 ^ns^	2.20 ^ns^	1.62 ^ns^
*p*-value	0.30	0.51	0.01	0.07	0.70	0.46	0.39	0.77

*p*-value indicates the probability of observing a difference as large or larger than that observed under the null hypothesis. Means followed by “ns” indicate that there are no significant differences in relation to the control (2 mL L^−1^ application on the leaf) or between the doses applied to the soil and the control dose (2 mL L^−1^ foliar application), according to Dunnett’s test (*p* < 0.05).

**Table 3:** Electrical conductivity of the soil saturation extract during the experimental conduction.

	**First Cycle**		
		**CEs (dS m^−1^)**	
**Doses (mL L^−1^)**	**Beginning of Cycle**	**End of Cycle**	
		**IPA-10**	**IPA-11**
0—Soil application		1.7 ^a^	1.6
1—Soil application		1.5 ^ab^	1.47
2—Soil application	0.85	1.5 ^ab^	1.48
3—Soil application		1.46 ^ab^	1.4
4—Soil application		1.2 ^b^	1.5
2—Application on the leaf		1.52 ^ab^	1.7
*p*-value	-	0.85	0.06
	**Second Cycle**		
		**ECs (dS m^−1^)**	
**Doses (mL L^−1^)**	**Beginning of Cycle**	**End of Cycle**	
		**IPA-10**	**IPA-11**
0—Soil application		4.70	4.69
1—Soil application		4.44	4.53
2—Soil application		4.46	4.57
3—Soil application	1.5	4.55	4.46
4—Soil application		4.56	4.44
2—Application on the leaf		4.49	4.48
*p*-value		0.34	0.29

ECs = Electrical conductivity of the soil saturation extract. *p*-value indicates the probability of observing a difference as large as or larger than that observed under the null hypothesis. Superscript letters indicate significant difference in probability by Tukey’s test (*p* < 0.01).

**Table 4:** Results of the physical and chemical analyses of the soil in the experimental area and the chemical analysis of the irrigation water used for the experimental cultivation of onion varieties, IPA-10 and IPA-11—Serra Talhada—April and August 2023.

Physical–Hydric Characterization of the Soil										
Depth	Ø		FC	Ds	Sand	Silt	Clay	EC-I	EC-II	
cm	%	% Weight		g cm^−3^		g kg^−1^		dS m^−1^		
0–30	48.0		14.0	1.39	809.24	105.69	85.07	0.85	1.50	
Chemical Characterization of the Soil During the First Cycle										
Depth (cm)		pH	O.M	V	H+Al	CTC	K^+^	Ca^2+^	Mg^2+^	Na^+^
			g kg^−1^	%	cmolc dm^−3^					
		6.7	8.8	75.1	1.8	7.3	0.54	3.7	1.21	0.04
0–30			P		2- S-SO_4_	Fe^2+^	Mn^2+^	Cu^2+^	Zn^2+^	B
			mg dm^−3^							
			57.8		4.56	85.0	46.4	1.4	1.4	0.32
Chemical Characterization of the Soil During the Second Cycle										
Depth (cm)		pH	O.M	V	H+Al	CTC	K^+^	Ca^2+^	Mg^2+^	Na^+^
			g kg^−1^	%	cmolc dm^−3^					
		7.4	6.6	99.7	0.02	9.28	0.88	5.03	3.13	0.23
0–30			P		2- S-SO_4_	Fe^2+^	Mn^2+^	Cu^2+^	Zn^2+^	B
			mg dm^−3^							
			93.0		3.0	143.6	68.0	1.78	2.6	0.64

Ø: Total porosity; Ds: soil density; CE-I: electrical conductivity in the first cycle; CE-II: electrical conductivity in the second cycle; CTC: cation exchange capacity; V: base saturation; O.M: organic matter (muffle method); FC: field capacity; H+Al: hydrogen + aluminum (potential acidity); K^+^: potassium; Ca^2+^: calcium; Mg^2+^: magnesium; Na^2+^: sodium; P: phosphorus; S: sulfur; Fe: iron; Mn^2+^: manganese; Cu^2+^: copper; Zn^2+^: zinc; and B: boron.

**Table 5:** Analysis of the water used for irrigating onions during the cultivation cycles from March to September 2023—Serra Talhada, PE, December 2023.

Properties of Irrigation Water										
EC	pH		Hardness-CaCO_3_		B		Cu	Mn	Fe	Zn
dS m^−1^	mg L^−1^									
1.68	6.63		610.5		0.129	0.019		0.04	<LQ	
Ca^2+^	Mg^2+^	K^+^	Na^+^	CO_3_^2−^	NaHCO_3_		Cl^−^	SO_4_^2−^	RAS	
mmolc L^−1^										
5.8	6.3	0.30	2.04	0.00	3.90		11.6	0.09	0.83	

EC: Electrical conductivity; K^+^: potassium; Ca^2+^: calcium; Mg^2+^: magnesium; Na^2+^: sodium; P: phosphorus; S: sulfur; Fe: iron; Mn^2+^: manganese; Cu^2+^: copper; Zn^2+^: zinc; B: boron; CaCO_3_: calcium carbonate; CO_3_^2−^: carbonate; NaHCO₃: sodium bicarbonate; Cl-: chloride; SO_4_^2−^: sulfate; RAS: sodium adsorption ratio; and <LQ: less than the qualification limit.

## Data Availability

Data are contained within the article.
